# Urothelial Plaque Formation in Post-Golgi Compartments

**DOI:** 10.1371/journal.pone.0023636

**Published:** 2011-08-24

**Authors:** Samo Hudoklin, Kristijan Jezernik, Josef Neumüller, Margit Pavelka, Rok Romih

**Affiliations:** 1 Faculty of Medicine, Institute of Cell Biology, University of Ljubljana, Ljubljana, Slovenia; 2 Department of Cell Biology and Ultrastructure Research, Center for Anatomy and Cell Biology, Medical University of Vienna, Vienna, Austria; The University of Kansas Medical Center, United States of America

## Abstract

Urothelial plaques are specialized membrane domains in urothelial superficial (umbrella) cells, composed of highly ordered uroplakin particles. We investigated membrane compartments involved in the formation of urothelial plaques in mouse umbrella cells. The Golgi apparatus did not contain uroplakins organized into plaques. In the post-Golgi region, three distinct membrane compartments containing uroplakins were characterized: i) Small rounded vesicles, located close to the Golgi apparatus, were labelled weakly with anti-uroplakin antibodies and they possessed no plaques; we termed them “uroplakin-positive transporting vesicles” (UPTVs). ii) Spherical-to-flattened vesicles, termed “immature fusiform vesicles” (iFVs), were uroplakin-positive in their central regions and contained small urothelial plaques. iii) Flattened “mature fusiform vesicles” (mFVs) contained large plaques, which were densely labelled with anti-uroplakin antibodies. Endoytotic marker horseradish peroxidase was not found in these post-Golgi compartments. We propose a detailed model of *de novo* urothelial plaque formation in post-Golgi compartments: UPTVs carrying individual 16-nm particles detach from the Golgi apparatus and subsequently fuse into iFV. Concentration of 16-nm particles into plaques and removal of uroplakin-negative membranes takes place in iFVs. With additional fusions and buddings, iFVs mature into mFVs, each carrying two urothelial plaques toward the apical surface of the umbrella cell.

## Introduction

Differentiation of the mammalian urothelium reaches its peak in the superficial cell layer, which consists of large umbrella cells [1], [2], [3]. Umbrella cells are unique for their luminal plasma membrane, as 70–90% of its area is covered by urothelial plaques [4], [5], [6]. The plaques are asymmetrically thickened membrane domains (also known as asymmetric unit membrane – AUM) with diameters of 600–1500 nm, separated by narrow rims of non-thickened membranes, called hinge regions [7], [8], [9], [10], [11]. The organization of uroplakins (UPs) in plaques [12], [13] defines their rigidity [14], [15] and is of major importance for the proper formation and maintenance of the urinary bladder's permeability barrier [16], [17], [18], [19].

Urothelial plaques are also present in fusiform vesicles (FVs), each containing two plaques [1], [11], [20], [21]. FVs function as transporting compartments for the delivery of urothelial plaques to the apical plasma membrane [22], [23], [24], [25], [26], [27], [28]. During bladder stretch, mechanoreceptors activate exocytosis of FVs by purinergic signalling, modulated by cAMP, Ca2, extracellular ATP, adenosine, the epidermal growth factor receptors and the actin cytoskeleton [29].

By that means, 25–55% of cytoplasmic FVs are incorporated into the apical plasma membrane [25], which makes its size increase, and urinary bladder can accommodate filling with urine [27], [30]. It has been shown that Rab 27b, Rab11a, syntaxin-1, SNAP-23 and synaptobrevin play important roles in apical targeting of FVs [31], [32], [33].

FVs may also be formed by endocytosis during contractions of the urinary bladder [25], [34]. Upon voiding, the redundancy of the apical plasma membrane is internalized and [1], [9] designated for degradation in lysosomes [35], [36], [37].

Two hypotheses have been put forward to explain a biosynthetic origin of urothelial plaques. According to the first one, it has been assumed that thickened membranes assemble in Golgi cisternae from the *cis*- to the *trans*- side, and eventually *trans*-cisternae mature into FV [8], [9], [38]. Finally, the FV detaches from the Golgi stack [1]. According to the second hypotheses [39], urothelial plaque assembly begins with the synthesis of four major uroplakins (UPIa, UPIb, UPII and UPIII) [12], [13], followed by the formation of UPIa/UPII and UPIb/UPIII dimers in the endoplasmic reticulum [40], [41], [42], [43], [44]. In the Golgi apparatus, two N-glycosylation sites on the UPII pro-sequence are converted into complex glycans [45], which results in the tethering of dimers into the heterotetramers (UPIa/UPII-UPIb/UPIII) [39]. It is assumed that by cleavage of the UPII pro-sequence into the *trans*-Golgi network, heterotetramers assemble into a 16-nm uroplakin particle [46] and particles are arranged into urothelial plaques in the post-Golgi compartments [5], [10], [47]. The maturation stages of urothelial plaques, following the exit of the heterotetramers out of the Golgi apparatus, have not been shown in umbrella cells.

In this study, we used for the first time a combination of uroplakin immunocytochemistry, freeze-fracturing and electron tomography to study the formation of urothelial plaques. We showed that plaques in the biosynthetic pathway form gradually in the individual post-Golgi compartments, termed: “uroplakin-positive transporting vesicles”, “immature fusiform vesicles”, and “mature fusiform vesicles”. Based on our data, we propose a novel model of urothelial plaque formation, which includes the fusion of post-Golgi compartments, sorting of uroplakin-positive membranes and removal of uroplakin-negative membranes.

## Materials and Methods

All animal experiments were approved by the Veterinary Administration of the Slovenian Ministry for Agriculture and Forestry (permission no. 34401-5/2009/4), and were in accordance with European guidelines and Slovenian legislation.

Urinary bladders of 6–8 weeks old male C57B6 mice, anaesthetized with CO_2_, were excised and immediately processed for microscopy. Five animals were sacrificed for studies of ultrastructure, 2 for each immuno-electron and immunofluorescence labelling study, and 3 for horseradish peroxidase internalization studies. At the time of sacrifice, the bladders contained less than 50 µl of urine, and were considered physiologically empty and contracted.

### Electron tomography

For high pressure freezing, bladders were removed immediately after death, tissue was impregnated with 6-hexadecene and cut into pieces with a diameter 2 mm and a thickness 200 µm. Samples were frozen with liquid nitrogen at 2100 bar in a Balzers HPM 010 apparatus. Freeze-substitution was done in the Automatic Freeze-Substitution System (EM AFS, Leica), following the protocol of Monaghan *et al* [48]. Briefly, samples were heated from −160 to −90°C (14 h, +5°C/h) and, at −90°C, acetone containing 2% OsO_4_ was introduced. Substitution/fixation was performed as: 8 h, −90°C; 6 h, +5°C/h; 8 h, −60°C; 6 h, +5°C/h; 8 h, −30°C. At −30°C, the acetone/OsO_4_ solution was replaced by fresh acetone and samples were then heated to +20°C (10 h, +5°C/h; 8 h, +20°C) and embedded in Epon.

Electron tomography was performed on 300 nm thick sections with a FEI Tecnai 20 TEM microscope, running at 200 kv. Tomograms covered angles between +65° and −65° in 1° steps. Micrographs were taken at a 6500× magnification, resulting in pixel size 2.72 nm. Modelling was done with Imod software (http://bio3d.colorado.edu/imod/). Projections of 3-D models of the Golgi apparatus and post-Golgi compartments are presented in the results section.

### Freeze-fracture

Urinary bladders were cut into <1 mm^3^ pieces and fixed in 4% PA+2% GA in 0.1 M cacodilate buffer for 3 hours at 4°C. After washing, samples were cryoprotected by incubation in 15% and 30% glycerol in 0.1 M cacodilate buffer, and frozen in liquid nitrogen. Fracturing was done on a freeze-etch machine Balzers BAF200 at −150°C. Exposed surfaces were shadowed with platinum at a nominal angle of 45° and further strengthened by carbon shadowed at a 90° angle. Replicas were transferred to room temperature, cleaned in 5% NaOH for 30 min at 70°C and in 10% ethanol for 20 min at room temperature. Replicas were put on copper microscopic grids and were examined with a Philips CM100 transmission electron microscope, running at 80 kV.

### Immuno-electron microscopy

Cryo-ultrathin sections were prepared by modified Tokuyashu method and labelled with anti-AUM rabbit polyclonal antibodies, generated against mature bovine uroplakins (a kind gift from Prof. T.T. Sun, University of New York, USA). Anti-AUM antibody reacts strongly with UPIIIa, moderately with UPIa and weakly with UPII [12]. Urothelium, cut into <1 mm^3^ pieces, was fixed in 4% PA+0.1% GA in 0.1 M phosphate buffer for 2 hours, washed in PBS, embedded in 12% gelatine and cryoprotected by incubation in 2.3 M saharose. Samples were then stored in liquid nitrogen. Samples were subsequently cut with a Leica FCS cryo-ultramicrotom at −120°C, and processed for immunolabelling. Non-specific labelling was blocked by 0.8% BSA, 0.1% fish gelatine and 5% fetal calf serum in PBS at room temperature for 30 min. Primary rabbit polyclonal antibodies against AUM (diluted 1∶10000) were incubated overnight at 4°C, washed in PBS, and incubated with goat anti rabbit secondary antibodies, conjugated with 10 nm colloidal gold (Sigma, diluted 1∶40), at room temperature for 1.5 h. Negative controls were done by omitting the primary antibodies, by incubation in rabbit serum, or by using inadequate primary antibodies. All cryo-ultrathin sections were examined with a Philips CM100 transmission electron microscope, running at 80 kV.

### Immunofluorescence labelling

Bladder whole-tissue urothelium was prepared as described previously [49]. Briefly, the bladder was cut into quarters, fixed in 4% paraformaldehyde in PBS, permeabilized with triton X-100 and immunolabelled with mouse IgG1 monoclonal anti-GM130 (BD Transduction Laboratories). Samples were then washed in PBS, incubated with AlexaFlour 488 goat anti-mouse secondary antibodies for 1.5 h, washed in PBS, and covered by VectaShield fluorescent mounting media containing DAPI. Prepared whole-tissue samples were mounted on glass slides with the apical surface of urothelium upwards, facing the lens.

For thin-section immunofluorescence labelling, urothelium was fixed as for immuno-electron microscopy and cut perpendicularly to the urothelial luminal surface into 300 nm thick sections using a Leica FCS cryo-ultramicrotom. Sections were incubated with monoclonal anti-giantin (BD Transduction Laboratories) and rabbit anti-AUM primary antibodies, followed by incubation with AlexaFlour 488 goat anti-mouse and AlexaFlour 488/555 goat anti-rabbit secondary antibodies. Negative controls were done by omitting the primary antibodies, by incubation in rabbit serum, or by using inadequate primary antibodies. Finally, slides were mounted with VectaShield (Vector laboratories) containing DAPI and visualized with a Nikon T300 or Zeiss AxioImager.Z1 microscope equipped with ApoTome upgrade.

### Horseradish peroxidase internalization

Mice were anaesthetized with ketamine-xylazine. In order to label the endocytotic membrane compartments involved in internalization of the apical plasma membrane, horseradish peroxidase (HRP, type II; SigmaAldrich) was instilled into the mouse urinary bladders. HRP was diluted in Krebs-Ringer medium (10 mg/ml) and 250 µl of this solution was slowly (over 2 minutes) instilled into the bladder with a syringe and catheter. After 2 hours, bladders were excised, thoroughly washed in cold Krebs-Ringer medium, cut into small pieces and fixed in 2.5% GA in 0.1 M cacodilate buffer. HRP was detected by DAB reaction. Samples were post-fixed in 1% OsO_4_ with 1% ferrocyanide in 0.1 M cacodilate buffer, dehydrated and embedded in Epon. Negative controls were done as above, except that HRP was omitted from the Krebs-Ringer medium. Ultrathin sections were observed on a transmission electron microscope, running at 80 kV.

### Quantitative analysis

Urinary bladders were sagitally divided into two halves; one half was prepared for ultrathin sections (see Electron tomography) and the other for freeze-fracture replicas (see Freeze-fracture).

Ultrathin sections (60 nm thick) were prepared from two Epon blocks of each animal and micrographs of 10 umbrella cells were taken at a magnification 21000× in a Philips CM100 microscope, running at 80 kv. We measured only the profiles of post-Golgi compartments that had membranes visible as two lipid layers around the whole compartment and that contained thickened membranes. By the first requirement, we ensured that vesicles were cut through their centre, and by the second that the analysed post-Golgi compartments contained urothelial plaques [2], [3], [50]. Measurements (twenty-five to fifty for each compartment) were done on a personal computer using ImageJ ver. 1.44 software for Windows [51]. The surface, intraluminal volume, and their ratio were calculated taking into consideration that membrane compartments are revolving geometric bodies and that the shape of UPTVs is limiting towards a sphere, iFV towards an ellipsoid, and the shape of mFVs is limiting towards a circular cylinder.

On freeze-fracture replicas, micrographs of umbrella cells were taken at a magnification 28500×. For analysis we only took into account vesicles that contained 16 nm particles [11], [14], [47]. Particle counting and surface measurements to calculate particle density (twenty to thirty for each compartment) were done using ImageJ software.

Statistical analyses were performed with Microsoft Office Excel 2007 software. Statistical significance was tested with Student's t-test.

## Results and Discussion

In our study, we analysed superficial urothelial cells that contained a well-developed Golgi apparatus, fusiform vesicles and strong anti-AUM immunolabelling reactions. We studied the membrane compartments in these cells, which participate in the biosynthesis of urothelial plaques that cover the apical plasma membrane. Based on the ultrastructure and immunolabelling, we showed that specific post-Golgi compartments, including “uroplakin-positive transporting vesicles” (UPTVs), “immature fusiform vesicles” (iFVs), and “mature fusiform vesicles” (mFVs), contribute to urothelial plaque formation.

### The Golgi apparatus is not the site of final plaque formation

The role of the Golgi apparatus (GA) in the process of urothelial plaque formation has been indicated by early morphological [8], [9], [38] and biochemical studies [39], [46]. We recently described the role of GA distribution and organization in the differentiation of urothelial cells [49]. Here we provide arguments that the GA is not the site of final plaque formation.

**Figure 1:**
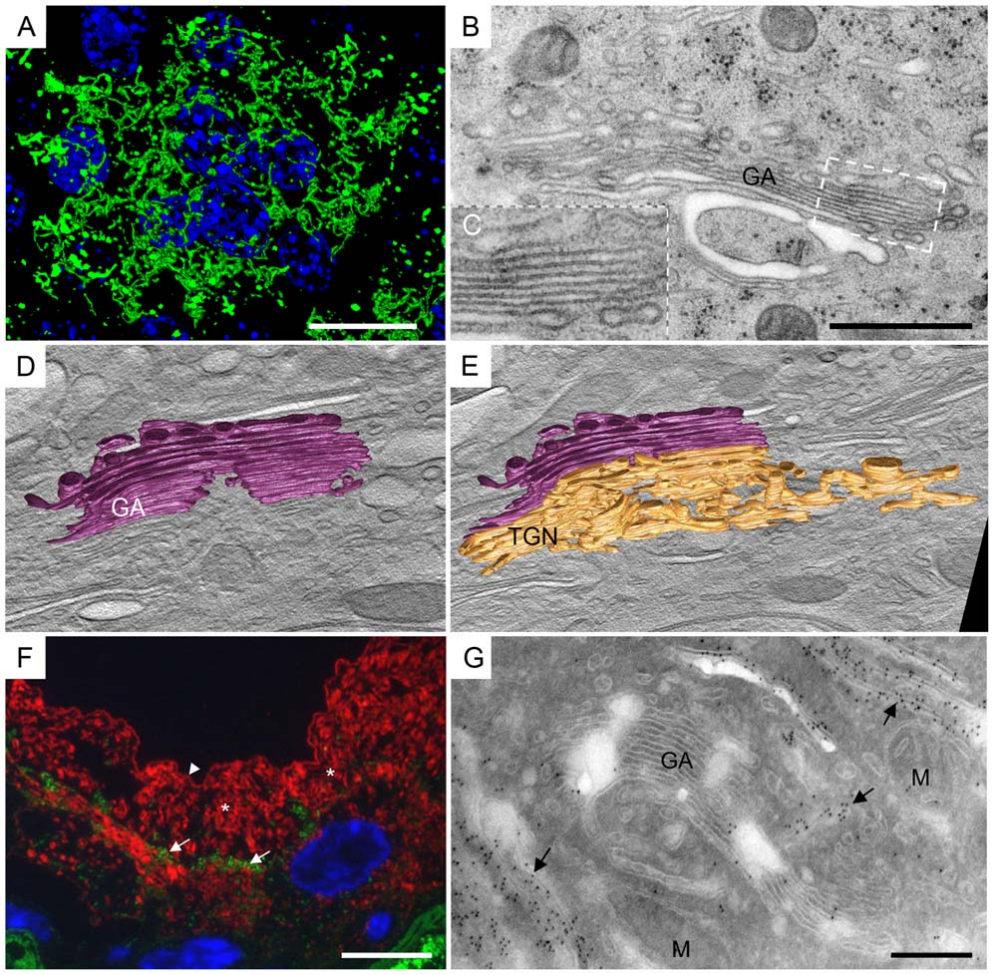
Uroplakins are not accumulated in the Golgi cisternae, but in the post-Golgi compartments. A) Top-view on the umbrella cell immunolabelled with anti-GM130 (green) shows a GA network spread across the whole cytoplasm. B) Plaques of asymmetric thickened membrane are not seen in the Golgi cisternae. C) Inset shows higher magnification of GA part, which has membranes 8–9 nm thick. D and E) Optical sections created from electron tomogram reconstruction, with superimposed models of the GA. D) GA forms flattened, occasionally fenestrated cisternae of various lengths (violet). E) From the rims of *trans*-Golgi cisterne and from the *trans*-Golgi network emanate tubulo-vesicular extensions (gold; GA cisternae are violet). F) Double immunolabelling with anti-gianti antibody (green, arrows) and anti-AUM antibody against mature uroplakins (red), shows no colocalization in the umbrella cell. GA (arrows) lies lateral to the nucleus, while uroplakins are seen throughout the cytoplasm (*) and on the apical surface (arrow-head). G) Labelling of cryo-ultrathin sections with anti-AUM antibody is negative in the GA and positive in post-Golgi compartments (arrows). Legend: M – mitochondria; blue – nucleus (DAPI). Bars 10 µm in A, F, 200 nm in B, G. Tomogram for D, E was taken at 6500×.

A top view of anti-GM130 immunolabelled umbrella cells revealed that the GA was spread over the central cytoplasm of umbrella cells and formed a network (Fig. 1A; negative controls were negative and are shown in Fig. S1A). That has been shown to be characteristic of the terminal differentiation of urothelial cells [49], [52]. By analysing the ultrastructure, a typical umbrella cell was shown to contain multiple interconnected GAs; an individual GA was composed of 5–9 aligned cisternae (Fig. 1B). Plaques of asymmetrically thickened membrane were not seen in the Golgi cisternae (Figs. 1B, C). Since thickened plaques were detectable in the post-Golgi compartments and on the apical plasma membrane, plaques would also be visible in the Golgi cisternae, if they were present.

A second argument against the GA as the site of final plaque formation came from electron tomography studies. Three-dimensional reconstructions by electron tomography revealed that Golgi cisternae were uniformly flattened in their centre (Fig. 1D). On the other hand, *trans*-cisternae and the *trans*-Golgi network (TGN) contained tubulo-vesicular extensions (rims; Fig. 1E, Videos S1, S2). The size and curvature of the extensions conflicts with the description of FVs, which are uniformly flat and never possess a significant intravesicular lumen. The 3-D reconstruction of the GA and surrounding compartments thus provides morphological evidence that Golgi cisternae do not directly mature into FVs [1], [8], [9], [38].

In order to see uroplakin particle accumulation in the GA, we used anti-AUM antibody, which is generated against total mature uroplakins. Double immunolabelling showed no co-localization of anti-AUM and anti-giantin antibodies (Fig. 1F; negative controls were negative and are shown in Fig. S1B). Although a section thickness of 300 nm provided high Z-axis resolution, we wanted to correlate the immunolabelling signal of the uroplakins with the ultrastructure of the GA. We labelled cryo-ultrathin sections with anti-AUM antibody, which again demonstrated no labelling of the Golgi cisternae (Fig. 1G; negative controls were negative and are shown in Fig. S1C). Labelling was observed in the tubulo-vesicular structures of the *trans*-Golgi network and in the post-Golgi compartments (Fig. 1G). Previous freeze-fracture studies have shown similar results; namely no accumulations of uroplakin particles in the GA [10]. Immunolabelling thus provides evidence that uroplakins become mature in the *trans*-Golgi network, as had been predicted by biochemical studies of uroplakin glycosylation [39], [46]; however, they are concentrated in mature plaques in the post-Golgi compartments.

### Uroplakin-positive transporting vesicles carry newly formed uroplakin particles

**Figure 2:**
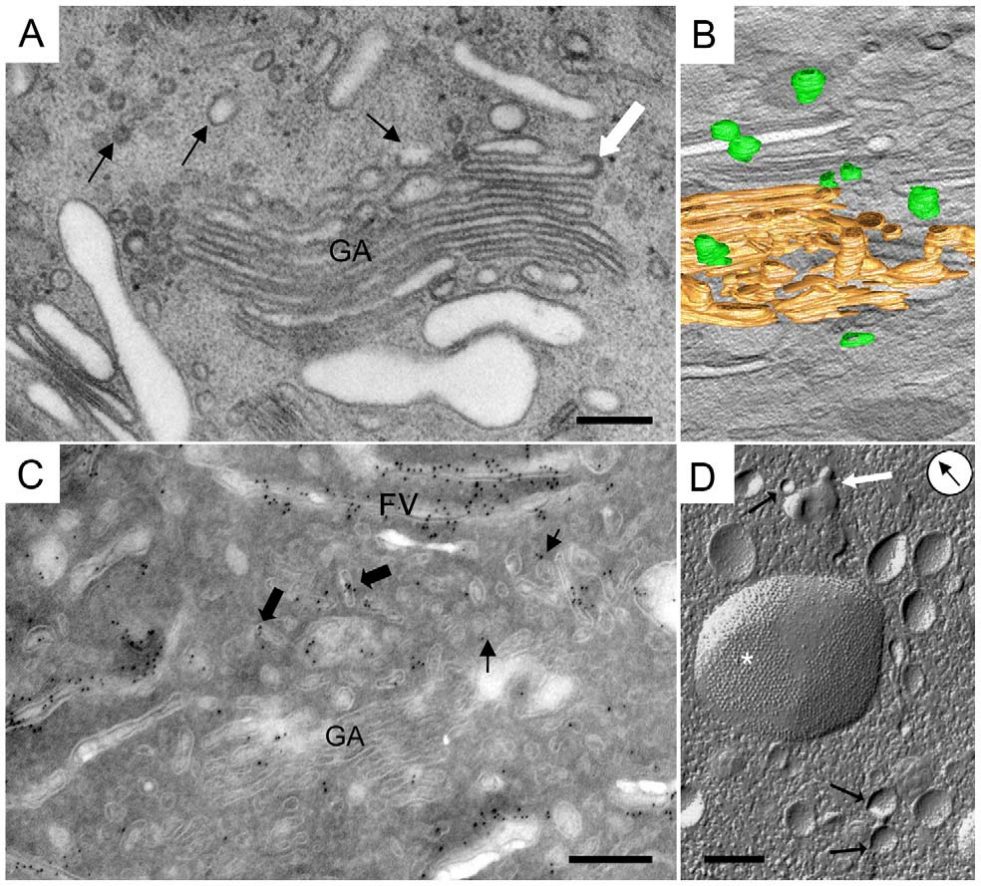
Mature uroplakins are first detected in UPTVs. A) UPTVs are post-Golgi compartments (black arrows), which are positioned next to the GA. Rims of some GA cisternae have coated membranes (white arrow). B) Projection of 3-D model shows that UPTVs are individual rounded membrane compartments (green; *trans*-Golgi network is gold). C) Sparse mature uroplakins are detected in UPTVs (arrows) and in the small iFVs (bigger arrows) next to the GA. Some iFVs are densely anti-AUM labelled. D) Freeze-fracture of umbrella cells shows UPTVs (arrows), which are positioned close to each other suggesting fusions of vesicles (white arrow). Larger iFVs carry numerous protein particles gathered into urothelial plaques (*). Arrow in circle indicates direction of Pt shadowing. Bars 200 nm.

Vesicles with an average diameter of 86 nm were noticed next to *trans*-Golgi cisternae (Fig. 2A, Table 1). Three-dimensional reconstructions disclosed that membrane compartments, which appeared as vesicles on thin sections, were distinct, spherical or slightly elongated (Fig. 2B, Videos S1, S2).

**Table 1:** Characteristics of post-Golgi compartments involved in the formation of urothelial plaques.

Class of vesicle	UPTVs	iFVs	mFVs
Major diameter (nm)	86 (9)	439 (201)§	958 (194)*
Surface∶ intraluminal volume ratio	0,129	0,078	0,423
Particles density in vesicles per µm^2^	414 (216)	550 (350)	1109 (398)*
Plaque ∶ non-thickened membranes ratio	-	2,73 (1,98)	8,78 (5,73)*
Plaque length ∶ circumference length, %	-	65 (17)	86 (8)*

Legend: in brackets - standard deviation,

§ - statistically significant UPTVs∶iFVs,

*- statistically significant iFVs∶mFVs.

Although urothelial plaques were not detected in these small vesicles, immunolabelling showed that some of them were anti-uroplakin positive (Fig. 2C). We therefore designated these post-Golgi compartments “uroplakin-positive transporting vesicles” (UPTVs). The low concentration of uroplakin particles in the membranes of UPTVs (414 per nm^2^) allow UPTVs to curve into small-diameter spherical shapes (Table 1). Such curving would be impossible with a high number of particles concentrated into mature plaques (1109 per nm^2^), which are rigid two-dimensional crystals [14], [15], [53]. Analysing the ultrastructure and freeze-fracture replicas of umbrella cells, UPTVs were observed close to each other (Fig. 2D) or close to iFV; this position suggests homo- and heterotypic fusions of membranes. Fusions of UPTVs would form bigger iFVs, carrying more uroplakin particles.

### Immature fusiform vesicles sort membranes and concentrate uroplakin particles

**Figure 3:**
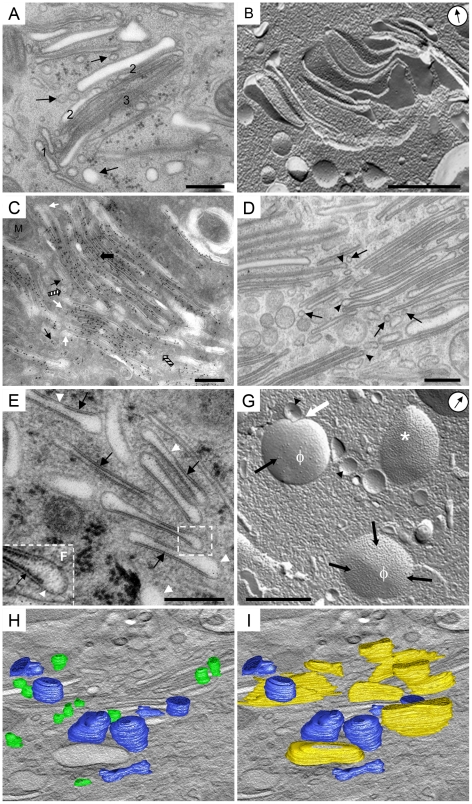
Spatial and size correlations between UPTVs, iFVs and mFVs suggest gradual formation of urothelial plaques in the biosynthetic pathway. A) UPTVs (arrows) and small iFVs (1) accumulate next to the polarised stack of larger iFVs (2) and mFVs (3). B) Freeze-fracture replica showing polarized stack of iFVs. Arrow in circle indicates direction of Pt shadowing. C) Uroplakin particles accumulate progressively from UPTVs, over iFVs to mFVs. UPTVs and smaller iFVs (black arrows) are detected next to larger iFVs. The central region of these vesicles shows heavy anti-AUM labelling (big arrow), while the rims remain uroplakin negative (white arrows). Note anti-AUM negative rounded vesicles (striped arrows) that might be involved in plaque formation by removing the excess of non-thickened membranes from maturing FVs. D) iFVs have vesicular dilatations (arrow-heads) and vesicles are seen in their vicinity (arrows), suggesting intense vesicular traffic . E) Various sizes of plaques of asymmetrically thickened membrane (black arrows) in iFVs are seen on ultra-thin section. Asymmetrically thickened membrane (12 nm thick) lines the central regions of iFVs, while non-thickened membranes (arrow-heads) line the rims of iFVs and separates plaques. Domains of non-thickened membrane appear dilated. F) At higher magnification, 12 nm thick plaque (arrow) and 9 nm thick hinge regions (arrow-head) are seen. G) Smaller iFVs (arrow-heads) and three larger iFVs, which contain a remarkably varied number of protein particles (black arrows) in their plaques. iFVs that contain a high number of particles (*) in their plaques, contain only a small amount of particle-free membranes (φ). White arrow shows close proximity of a smaller and bigger iFV, a position suggesting their fusion, thus increasing the number of uroplakin particles. H and I) Optical sections created from electron tomogram reconstruction, with superimposed models of iFVs. H) UPTVs are positioned close to each other (green), and next to small iFV (blue). I) Small iFVs (blue) are spherical or ellipsoid, while bigger iFVs (yellow) are flattened. Bars 200 nm, 500 nm in B, G.

The term FV is used to describe dilated spherical-to-discoidal or flattened vesicles with asymmetrically thickened membranes [4], [9], [16], [19], [29], [32], [49], [50], [52], [54], [55]. In our study, we distinguished two sub-populations of FVs: the first were termed “immature FVs” (iFVs), and the second as “mature FVs” (mFVs). iFVs were observed as a spectrum of 100 to 800 nm long, dilated to flattened vesicles, containing various lengths of morphologically recognizable plaques of AUM (Fig. 3). iFVs represent the sub-population of vesicles that have been described elsewhere by their assortment of profiles, e.g., dilated, spherical or discoidal vesicles [4], [5], [8], [19], [29], [50]. Comparing those vesicles with mFVs, it was evident that iFVs had a smaller overall size (average 439 vs. 958 nm), shorter length of plaques (average diameter 327 vs. 851 nm, occupying 65 vs 86% of the circumference length) and larger intraluminal volume (surface: intraluminal volume ratio 0,078 vs 0,423; Tables 1, S1), which shows that iFVs are distinct post-Golgi compartments. In order to distinguish iFVs from mFVs, we recommend the term iFVs when referring to the population of heterogeneous membrane compartments with a central role in plaque formation; namely, intensive membrane adding, sorting and concentration of the uroplakin particles, as described below.

Adding uroplakin particles to iFVs is carried out by UPTVs. UPTVs in the GA region were close to each other and, next to them, small, individual iFVs were observed (Figs. 3A, H). Larger iFVs were flattened and were typically arranged in stacks of 3 to 10 vesicles, positioned parallel to each other (Figs. 3A, B, I, Videos S1, S2). Stacks were polarized in the sense that they incorporated smaller, dilated vesicles at one side, and larger, uniformly flattened vesicles at the other side of the stack (Figs. 3A, D). The intensity of anti-AUM labelling increased in larger, stacked iFVs compared to UPTVs and smaller iFVs (Fig. 3C). The rims of stacked iFVs were made of non-thickened membranes, which were uroplakin-negative (Fig. 3C) and frequently possessed dilatations (Fig. 3D). The diameter of the dilatations was approximately 100 nm, which corresponded to the size of UPTVs and to small, uroplakin-negative vesicles that were also found next to the GA. That implies that fusions of UPTVs with iFVs, and the detachment of uroplakin-negative vesicles from them, could be a mechanism of uroplakin particle delivery to the growing urothelial plaque. An alternative way of obtaining larger plaques would be direct membrane connections between the GA and iFVs, by which uroplakin particles would move. We employed electron tomography to test this hypothesis, which showed no direct connections between the GA and iFVs (Figs. 3H, I).

Sorting urothelial plaques takes place in 2 regions of iFVs: the centre and rims. Results showed that plaques occupied the central parts of iFVs, while their rims were composed of non-thickened membranes (Figs. 3E, F). Plaques opposed each other at the parallel sides of the vesicle; however, the sides of the vesicle were not identical. There were differences in plaque lengths and in plaque number between the sides of the vesicles, i.e., one side contained a few smaller plaques, separated by non-thickened membranes, while the other side had a single large plaque (Fig. 3E). These observations were further supported by analysing freeze-fracture replicas, whereby it was noted that the diameter of the plaque area (327±203 nm) and the number of uroplakin particles (550±350 per µm^2^ ) varied also among iFVs of the same size (Fig. 3G, Table 1, S1); the same was observed by Severs and Hicks [10], and provides further evidence of the progressive maturation of urothelial plaques in iFVs.

The heterogeneity of shape and size of iFVs correlated with the amount of thickened membranes they contained (Tables 1, S1). The more non-thickened membranes a vesicle contained, the smaller and more spherical was its appearance, and vice-versa, the larger the plaques contained in a vesicle, the larger and more flattened was its appearance (Table 1, S1). That observation is in agreement with the finding that uroplakin particle concentration and the size of the urothelial plaque are determined by the force equilibrium of head-to-head interactions between neighbouring uroplakin particles and local surface tensions [14]. Uroplakin particles are thereby progressively added to iFVs and aggregate until the final size of the urothelial plaque is reached in mFVs.

These results support the hypothesis that iFVs are the site of intensive membrane sorting and concentration of uroplakin particles. This means that iFVs are a post-Golgi compartment, which has a central role in plaque formation. Compartments that succeed iFVs, i.e., mFVs, have a role in the transport of mature plaques between cytosol and the apical plasma membrane.

### Mature fusiform vesicles are the final stage of plaque formation

**Figure 4:**
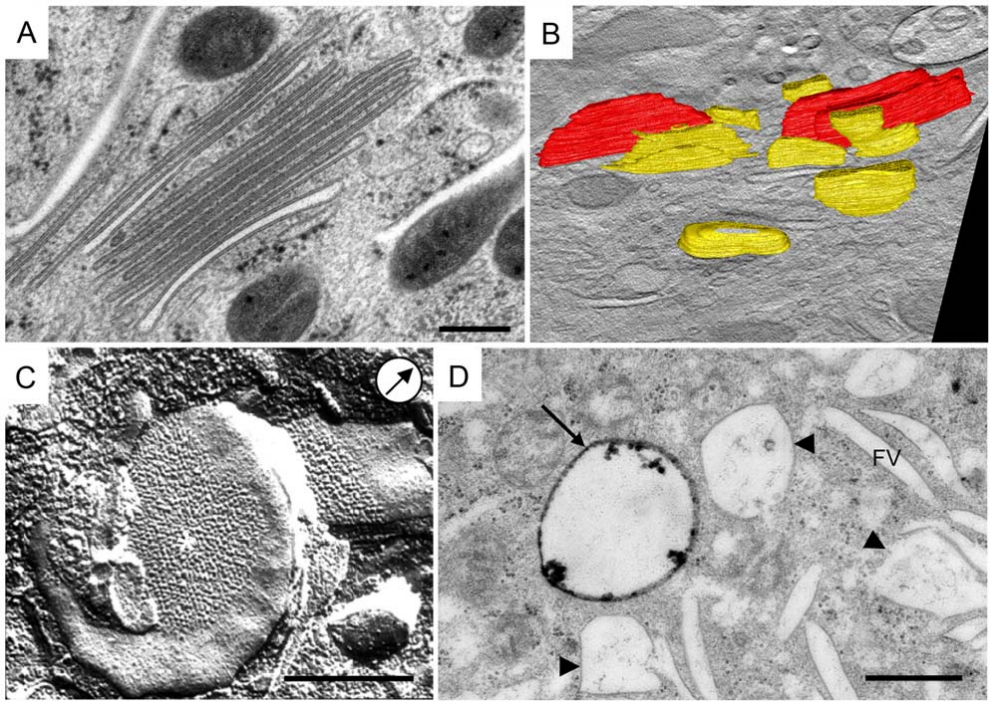
mFVs present the final stage of urothelial plaque formation. A) On the micrograph are shown mFVs organized into a stack. mFVs have a narrow intravesicular lumen and un-dilated rims. B) Projection of 3-D model shows difference in size and shape between bigger iFV (yellow) and mFVs (red). C) mFVs have urothelial plaques (*) positioned centrally. Note flattened, coin-like shape of the mFV. Arrow in circle indicates direction of Pt shadowing. D) After two hours of endocytosis, the HRP reaction product is present in a multivesicular body (arrow), while the majority of multivesicular bodies (arrow-heads) and all of the FVs remain un-labelled. Bars 500 nm.

mFVs were the most abundant membrane compartments of umbrella cells that were investigated in our studies. The presence of mFVs was evidence that cells are terminally differentiated and therefore represent typical umbrella cells of the bladder [3], [19]. The mFVs analysed here were on average ≈950 nm long, and in sectional profile uniformly flattened, coin-like vesicles (Tables 1, S1, Fig. 4A).

mFVs were gathered into stacks (Figs. 4A) or they were found as individual compartments. They have a high surface-to-volume ratio (Table 1). The surface of an average mFV had increased by 480% compared to an iFV, while its intraluminal volume simultaneously decreased by 13% (Table S1).

In agreement with the literature [1], [19], [29], [50], observed mFVs contained two urothelial plaques, connected by a non-thickened ‘hinge’ region at their rims (Fig. 4A). Each plaque consisted of hexagonally ordered 16 nm urothelial particles (Fig. 4C). Quantitative analysis showed that the size of a plaque was on average 2,6 times bigger, and the number of particles per plaque was on average 2 times higher, in mFVs than in iFVs (Tables 1, S1). This supports the idea that mFVs represent the final stage of plaque formation.

### The majority of urothelial plaques are of biosynthetic origin

Urothelial plaques can origin from *de novo* formation in the biosynthetic pathway or from internalization of the apical plasma membrane [25], [34]. In order to distinguish membrane compartments of the biosynthetic pathway from those of the endocytotic pathway, we analysed umbrella cells after HRP instillation into the urinary bladder lumen. After two hours of endocytosis from the apical plasma membrane, HRP-reaction products were found only sparsely inside the cells. As has also been observed elsewhere [37], [56], only some small rounded vesicles in the subapical region and multivesicular bodies were labelled with HRP-reaction products (Fig. 3D; negative controls were negative and are shown in Fig. S1D). HRP-reaction products were not detected in iFVs, mFVs or in any structure in the GA region. This is in agreement with studies of Kreft *et al* [52], which show that the rate of endocytosis from the apical plasma membrane decreases as urothelial cells advance in their differentiation, and reaches its minimum in the terminally differentiated umbrella cells.

Our study therefore shows that the majority of urothelial plaques are of biosynthetic and not endocytotic origin.

### Conclusion

**Figure 5:**
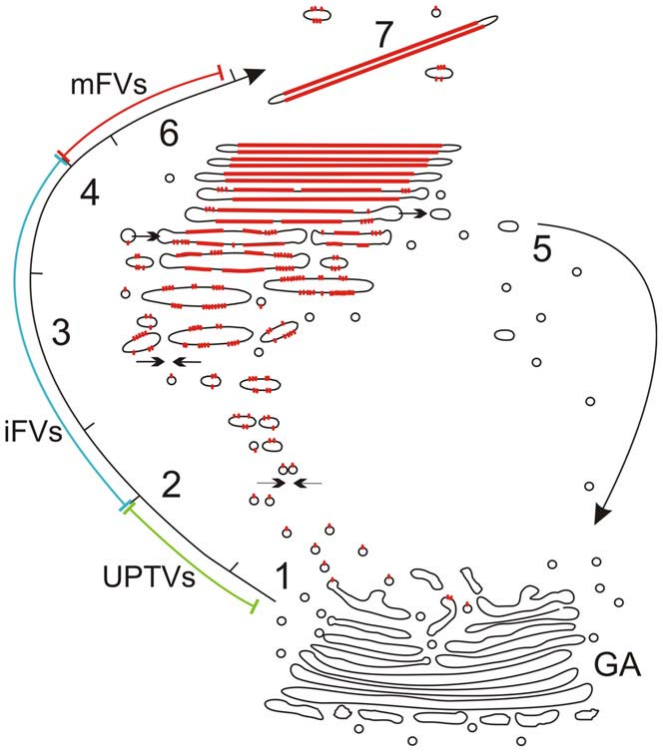
Model of post-Golgi formation of urothelial plaques. 1) UPTVs with sparse uroplakin particles (red) leave trans-Golgi network (GA). 2) iFVs are formed by homotypic fusion of UPTVs (arrows). 3) In iFVs, the number of particles increases and small plaques are formed, which leads to a flattening of the iFVs. Small iFVs further fuse with each other (arrows) or with UPTVs to form large iFVs that eventually constitute a polarized stack of iFVs. 4) In iFVs membrane sorting and particle concentration take place. At the vesicle's rims, fusions with UPTVs and iFVs incorporate additional uroplakin particles (arrow in), while budding removes excess of non-thickened membrane (arrow out and 5). In the central part of the vesicle, uroplakin particles are concentrated into growing plaque (red line). 6) When the size of the plaque reaches dynamic equilibrium [14], mFVs are formed. 7) mFVs are detached from the stack and can be transported to the apical plasma membrane. Legend: green, blue and red lines represent initial-, central- and final stage of plaque formation, characterized by UPTVs, iFVs and mFVs, respectively.

Umbrella cells, as highly specialized non-secreting epithelium [1], are a unique *in vivo* model for studies of membrane formation, maturation and the dynamics of polarized traffic [19], [21], [29], [50]. We have here combined microscopical methods to gain insights into the formation of urothelial plaques in mouse umbrella cells. Our results indicate that urothelial plaques have a predominantly biosynthetic origin. The GA is the site of uroplakin glycosylations, their conformation changes and tetramerization [39]; however, it is not the site of plaque formation. Instead, we propose an improved model (Fig. 5) of progressive plaque formation in three distinct post-Golgi compartments: i) “uroplakin-positive transporting vesicles” transport individual uroplakin particles and are fused into “immature fusiform vesicles” (initial stage of plaque formation). ii) In immature fusiform vesicles, uroplakin particles are concentrated into plaques and uroplakin-negative membranes are removed (adding, sorting and concentrating membranes; the central stage of plaque formation). iii) When the urothelial plaque reaches its final size, immature fusiform vesicles are transformed into “mature fusiform vesicles” (final stage of plaque formation) and stored urothelial plaques can be transported into the apical plasma membrane of umbrella cells.

## Supporting Information

Figure S1 **Negative controls.** A) Negative control for anti-GM130 immunolabelling. No green color (anti-GM130) is seen in umbrella cells. B) Negative control for anti-giantin and anti-AUM immunolabelling. No green (anti-giantin) or red (anti-AUM) color is seen in umbrella cells. C) Negative control for anti-AUM immunolabelling on cryo-ultra thin sections. No gold particles on mFV or on the apical plasma membrane of umbrella cells (arrow). D) Negative control for HRP internalization experiment. Note, no black HRP-reaction products are seen on the apical plasma membrane or in the cytoplasm of umbrella cells. Legend: blue – nucleus (DAPI). Bars: 10 µm in A, B, 500 nm in C, D.
(TIF)

Video S1 **Three-dimensional reconstruction of Golgi apparatus and post-Golgi compartments in the umbrella cell.**
(WMV)

Video S2 **Three-dimensional model of Golgi apparatus and post-Golgi compartments involved in the formation of urothelial plaques in the umbrella cell.**
(WMV)

Table S1 **Characteristics of post-Golgi compartments involved in the formation of urothelial plaques (extended).**
(DOCX)
