# Supplementary figures and images for: Urothelial Plaque Formation in Post-Golgi Compartments
Source: PLoS One. 2011 Aug 24;6(8):e23636. doi: 10.1371/journal.pone.0023636 (PMC3161059; doi:10.1371/journal.pone.0023636)

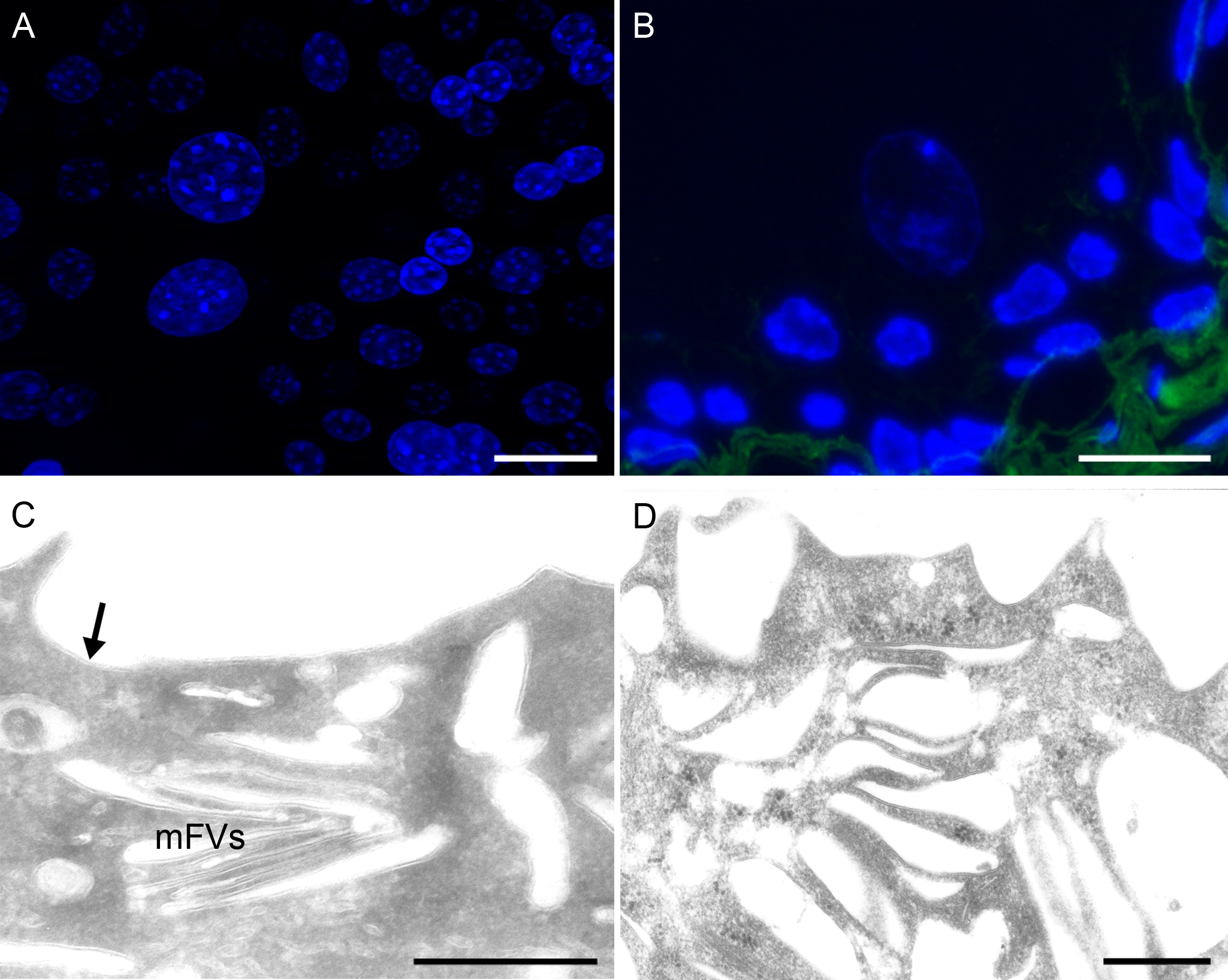

Supplement: Figure S1 — Negative controls. A) Negative control for anti-GM130 immunolabelling. No green color (anti-GM130) is seen in umbrella cells. B) Negative control for anti-giantin and anti-AUM immunolabelling. No green (anti-giantin) or red (anti-AUM) color is seen in umbrella cells. C) Negative control for anti-AUM immunolabelling on cryo-ultra thin sections. No gold particles on mFV or on the apical plasma membrane of umbrella cells (arrow). D) Negative control for HRP internalization experiment. Note, no black HRP-reaction products are seen on the apical plasma membrane or in the cytoplasm of umbrella cells. Legend: blue – nucleus (DAPI). Bars: 10 µm in A, B, 500 nm in C, D. (TIF) [file pone.0023636.s001.tif]
