# Supplementary material for: Urothelial Plaque Formation in Post-Golgi Compartments
Source: PLoS One. 2011 Aug 24;6(8):e23636. doi: 10.1371/journal.pone.0023636 (PMC3161059; doi:10.1371/journal.pone.0023636)
Supplement: Table S1 — Characteristics of post-Golgi compartments involved in the formation of urothelial plaques (extended). (DOCX) [file pone.0023636.s004.docx]

**Table S1:** Characteristics of post-Golgi compartments involved in the formation of urothelial plaques (extended).

| **Class of vesicle** | **UPTVs** | **iFVs** | **mFVs** | **t-test** | |
| --- | --- | --- | --- | --- | --- |
|  |  |  |  | UPTVs : iFVs | iFV : mFV |
| Major diameter of the profile (nm) | 86 (9) | 439 (201) | 958 (194) | 2,185∙10^-7^* | 1,58∙10^-12^* |
| Minor diameter of the profile | 86 (9) | 61,1 (23,1) | 28,8 (4,7) | 8,69∙10^-4^* | 5,68∙10^-8^* |
| Diameter of plaques (nm) | - | 327 (203) | 851 (208) | - | 3,15∙10^-12^* |
| Length of non-thickened membranes (nm) | - | 138 (50) | 133 (79) | - | 0,7870 |
| Plaque : non-thickened membranes ratio | - | 2,73 (1,98) | 8,78 (5,73) | - | 2,86∙10^-7^* |
| Plaque length : circumference length, % | - | 65 (17) | 86 (8) | - | 1,06∙10^-5^* |
| Calculated surface of the average compartment (nm^2^) | 23235 | 321372 | 1525890 |  |  |
| Calculated intraluminal volume of the average compartment (nm^3^) | 179594 | 4137247 | 3604050 |  |  |
| Particles density in vesicles ( µm^2^) | 414 (216) | 550 (350) | 1109 (398) | 0,2489 | 9,81∙10^-4^* |
| Particles density in plaque (no. : µm^2^) |  |  | 1903 (750) |  |  |

Legend: in brackets - standard deviation, * - statistically significant (p<0,01)
